# Radiographic diagnosis of a catheter rupture in a ventriculoperitoneal shunt following head trauma in a dog

**DOI:** 10.1111/jsap.13802

**Published:** 2025-01-15

**Authors:** L. Martin Garcia, R. Gutierrez‐Quintana, V. Gonzalo Nadal, A. Cloquell Miro

**Affiliations:** ^1^ Neurology Department University of Glasgow Glasgow UK

**FIG 1:**
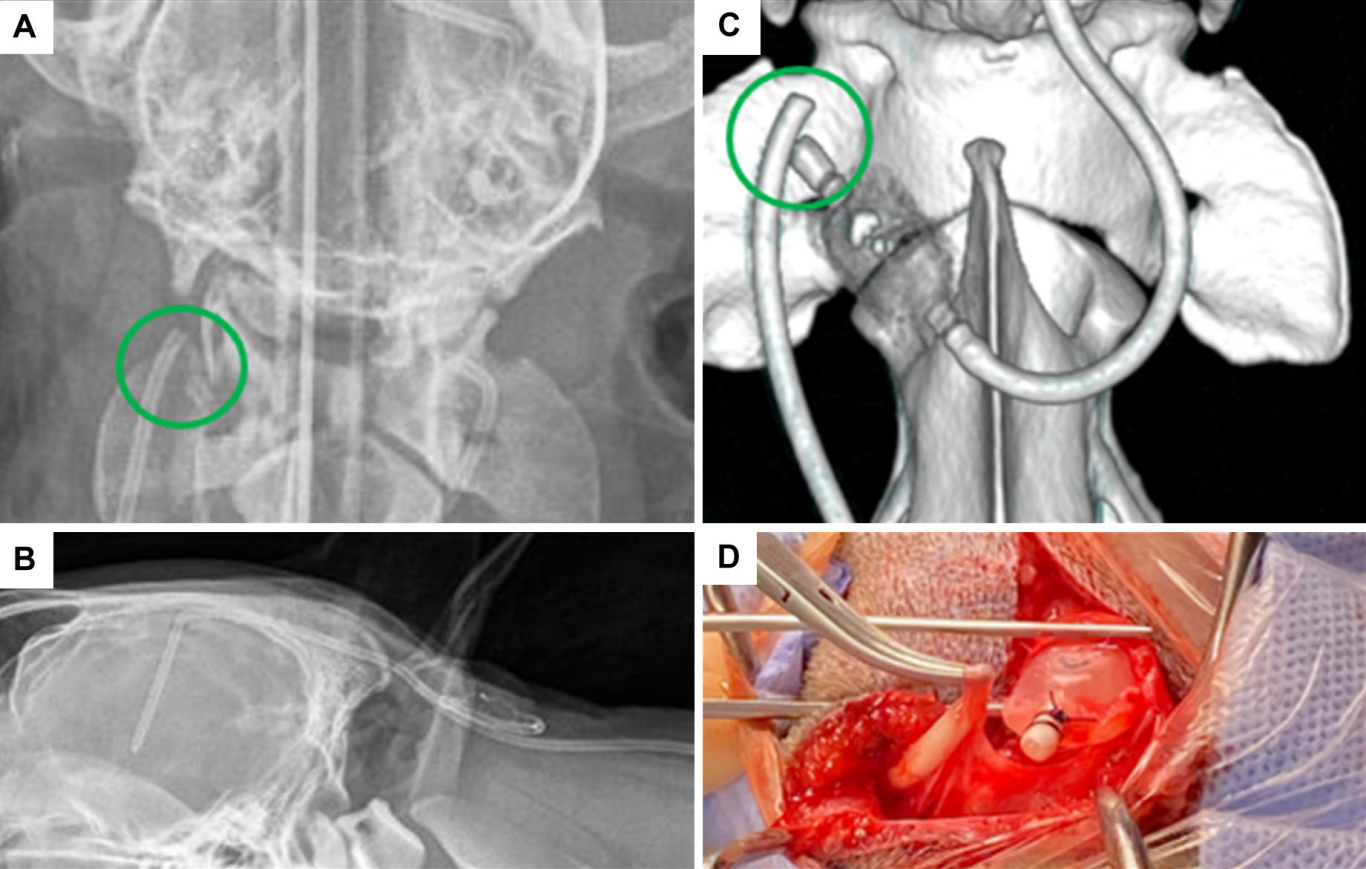
(A) Dorsoventral radiographic image of atlantooccipital region reveals a discontinuation in the ventriculoperitoneal shunt (VPS) catheter (green circle) situated over the right transverse process of C2. (B) Right lateral radiograph of the same anatomical location demonstrates poor visualisation of the shunt rupture. (C) Three‐dimensional computed tomography reconstruction image of the cranial cervical area provides clearer visualisation of the catheter rupture (green circle). (D) Intraoperative image of the patient verifies the presence of catheter rupture and intact sutures connecting the cranial end of the distal catheter to the VPS valve.

Four‐year‐old male neutered Border Collie presented with a 3‐month history of proprioceptive ataxia, low head carriage, delayed postural reactions on thoracic limbs and neck pain. The patient was diagnosed with congenital obstructive hydrocephalus with secondary syringohydromyelia and underwent a successful ventriculoperitoneal shunt (VPS) surgery. A year after surgery, the dog presented with the same neurological signs 2 weeks after suffering head trauma. On manual palpation, the VPS valve was firmly filled and non‐compressible suggesting the presence of an occlusion distally to the valve. Right lateral and dorsoventral radiographic images of the neck, thorax and abdomen were obtained to assess the integrity of the shunt catheters and valve. Dorsoventral images showed a rupture of the distal catheter proximally to the valve, which was easily missed in the lateral views (Fig 1A, B). Computed tomography confirmed the radiological findings (Fig 1C), and no other abnormalities were noted. The patient underwent revision surgery (Fig 1D), and the affected part of the shunt catheter and the valve were replaced. The patient recovered successfully and 1 month after surgery, neurological examination demonstrated mild proprioceptive ataxia, with the rest of the signs resolved. As previously described in the veterinary literature, complications following VPS placement include ventricular catheter migration, infection, kinking of the catheter, disconnection between the catheters and the valve, under or excessive drainage and abdominal skin necrosis. To the author's knowledge, this is the first case of VPS rupture following head trauma, which should be considered in patients with a history of head trauma after shunt placement who present with relapse of neurological signs. This case emphasises the importance of performing orthogonal radiographic views of the shunting route, and the potential use of the manual evaluation (by pumping) of the valve reservoir for testing VPS sufficiency.

